# Spontaneous Development of Full Weight-Supported Stepping after Complete Spinal Cord Transection in the Neonatal Opossum, *Monodelphis domestica*

**DOI:** 10.1371/journal.pone.0026826

**Published:** 2011-11-02

**Authors:** Benjamin J. Wheaton, Jennifer K. Callaway, C. Joakim Ek, Katarzyna M. Dziegielewska, Norman R. Saunders

**Affiliations:** Department of Pharmacology, The University of Melbourne, Parkville, Victoria, Australia; University of Western Ontario, Canada

## Abstract

Spinal cord trauma in the adult nervous system usually results in permanent loss of function below the injury level. The immature spinal cord has greater capacity for repair and can develop considerable functionality by adulthood. This study used the marsupial laboratory opossum *Monodelphis domestica*, which is born at a very early stage of neural development. Complete spinal cord transection was made in the lower-thoracic region of pups at postnatal-day 7 (P7) or P28, and the animals grew to adulthood. Injury at P7 resulted in a dense neuronal tissue bridge that connected the two ends of the cord; retrograde neuronal labelling indicated that supraspinal and propriospinal innervation spanned the injury site. This repair was associated with pronounced behavioural recovery, coordinated gait and an ability to use hindlimbs when swimming. Injury at P28 resulted in a cyst-like cavity encased in scar tissue forming at the injury site. Using retrograde labelling, no labelled brainstem or propriospinal neurons were found above the lesion, indicating that detectable neuronal connectivity had not spanned the injury site. However, these animals could use their hindlimbs to take weight-supporting steps but could not use their hindlimbs when swimming. White matter, demonstrated by Luxol Fast Blue staining, was present in the injury site of P7- but not P28-injured animals. Overall, these studies demonstrated that provided spinal injury occurs early in development, regrowth of supraspinal innervation is possible. This repair appears to lead to improved functional outcomes. At older ages, even without detectable axonal growth spanning the injury site, substantial development of locomotion was still possible. This outcome is discussed in conjunction with preliminary findings of differences in the local propriospinal circuits following spinal cord injury (demonstrated with fluororuby labelling), which may underlie the weight bearing locomotion observed in the apparent absence of axons bridging the lesion site in P28-injured *Monodelphis*.

## Introduction

The immature spinal cord, compared with adult, has a much greater ability to repair itself following injury and can subsequently develop considerable functionality. This has been well documented in marsupial species, which are born at a very early stage of brain and spinal cord development allowing surgical intervention *ex utero* at times corresponding to E14–16 in rodents [1], [2]. Thus in the South American laboratory opossum, *Monodelphis domestica*, if the spinal cord *in vitro* is completely transected in the first week of life there is a profuse growth of axons across the site of injury [3]–[5]. This has also been demonstrated *in vitro* in fetal rat spinal cord [2]. The same profuse growth of axons across a complete spinal transection *in vivo* in *Monodelphis domestica* was subsequently demonstrated together with the finding, which could not be investigated using *in vitro* preparations, that as the animals grow their locomotor development is substantially normal [6], [7]. Similar findings have been reported for the North American opossum, *Didelphis virginiana* [8].

The ability of lesioned axons to regenerate and grow across a site of transection appears to be diminished in more mature animals and depends on the brainstem nuclei of origin of the axons [9]. The upper age limit for a regenerative response of spinal cord axons in either species of opossum has not been determined. Wang et al. [10] found evidence of axonal growth across a transected spinal cord for some supraspinal axons at least until P26.

Retrograde labelling studies in *Monodelphis* showed that following transection made at P7 approximately 50% of the lesioned axons originating from brainstem nuclei had regenerated [11]. Similar results have also been reported for *Didelphis* [9]. However, at the time when the lesions were made, the spinal cord was still growing and many additional axons crossed the site of injury as part of normal development. Thus by adulthood the proportion of regenerated axons in the total number of axons crossing the lesion site was estimated to have been about 5% [11].

Wang et al. [8] showed that *Didelphis virginiana* with spinal cord transections in the first week of life demonstrated near normal locomotor function as adults as assessed by the open-field BBB locomotor rating scale [12]. When these animals were re-transected at the same site when adult and after they had recovered from transient initial paralysis they demonstrated considerably better BBB scores than animals transected for the first time when adult. By 2 weeks after re-transection their movements included partial weight bearing by the hindlimbs and some degree of uncoordinated limb movements. These studies suggested, firstly, that at least some of the regrowing descending axons were functional and, secondly, that the circuitry in the spinal cord had also undergone some remodelling.

Sparing of function or better functional recovery in the absence of regeneration following complete spinal cord transection in immature compared with adult lesioned animals has been described in neonatal cats [13], [14] and rats [15], [16]. This has been attributed to (i) abnormal growth of dorsal root axons and/or propriospinal and interneuronal connections caudal to the transection after it had been made [17], (ii) immaturity of descending systems at the time of transection, described in neonatal cats [14] or (iii) lack of inhibition within the segmental systems in the immature spinal cord, using newborn cats as a model [13]. However, these early studies were handicapped by the lack of adequate methods for tracing supraspinal or local neural pathways.

In the present study we have used postnatal opossums (*Monodelphis domestica*) and made a complete spinal cord transection at an age (P7) when profuse growth of axons occurs across the lesion [11], [18], with substantially normal locomotor development by adulthood [1], [6], [7]. These animals have been compared with those of an age (P28) when no re-growing axons could be demonstrated. The opossum provides a good system in which to study the differences in axon growth and locomotor development following early spinal cord lesions made at different ages because these animals are born so early in central nervous system development that the whole study could be completed postnatally. To examine a stage when axon growth occurs following a spinal cord transection in rodents would require *in utero* operations [2], [19]. Our results of the present study show that although in P28 lesioned animals no supraspinal axon growth through the site of injury could be demonstrated, full weight supported stepping was observed. In the P7 lesioned animals when tested as adults, there was a lack of correlation between the behavioural scores and the numbers of back-labelled brainstem neurons or volume of tissue growing at the site of injury. The possible contribution of remodelling of local neural circuits to behaviour following injury is discussed.

## Materials and Methods

### Ethics statement

All animal experiments were conducted following National Health and Medical Research Council guidelines and were approved by the University of Melbourne Animal Ethics Committee, Ethics #0707108.

### Animal Husbandry

**Figure 1:**
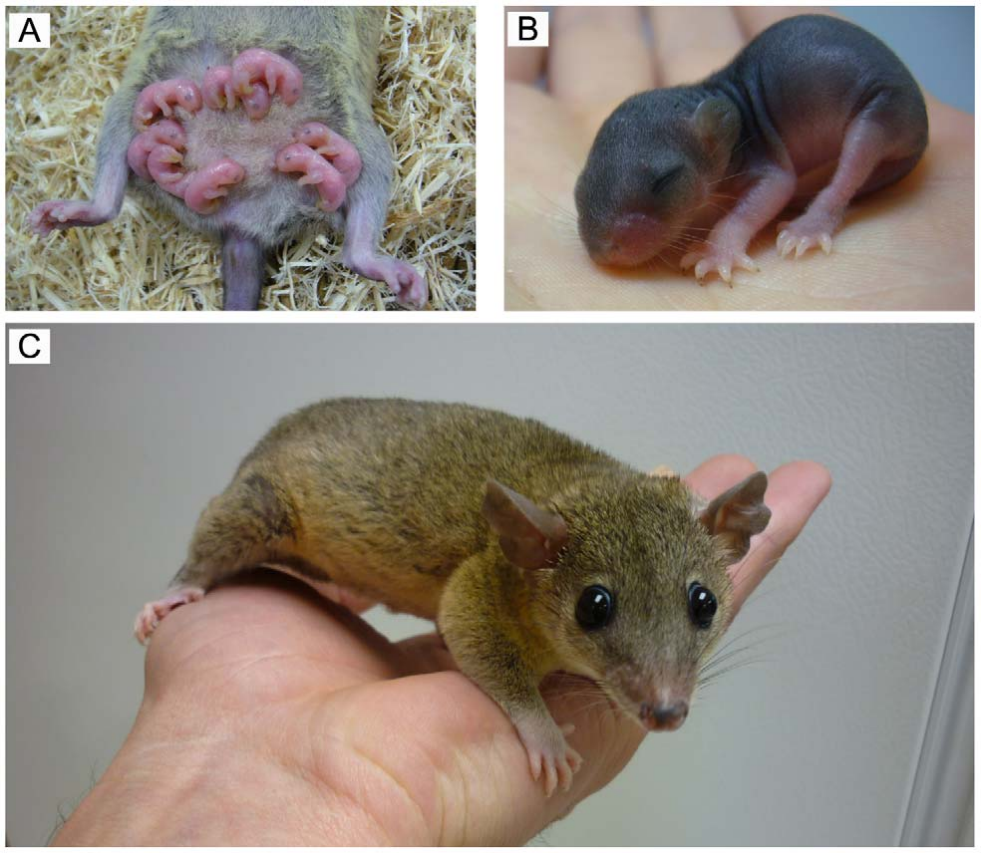
The Grey Short-tailed Opossum (*Monodelphis domestica*). ***A:*** Opossum pups at P7, attached to the mother's abdomen; ***B:*** Opossum pup at P28; ***C:*** Fully grown opossum.

A South American grey short-tailed laboratory opossum, *Monodelphis domestica*, was used in this study (see Fig. 1). All opossums were obtained from the *Monodelphis* colony maintained by the Biological Research Facility at the University of Melbourne. Full descriptions of care and breeding for this species have been published previously [1], [20]–[22]. Briefly, mothers with pups are housed in polycarbonate boxes with additional nesting material in the animal facility. Up to about postnatal day (P) 15 opossum pups are attached permanently to the mother's teats, after which they detach for increasing lengths of time until they are weaned at P60–65. Food and water are given *ad libitum*. All animals are kept in a temperature- and humidity-controlled environment (27°C; 60% humidity) with a 14:10 hour light/dark cycle.

All experiments were approved by The University of Melbourne Animal Ethics Committee. All animals undergoing surgical manipulation were anaesthetised to a surgical level and all procedures were carried out under sterile conditions. Potential post-operative pain was managed using intraperitoneal (i.p.) injections of Buprenorphine (0.6 mg/kg). No signs of procedure-related infection in any of the injured pups were observed, but in a few cases spinal-injured opossums tended to chew at desensitised hind paws. Infection of a damaged foot occurred in one case. This was treated using topical applications of Fuciderm (fusidic acid, betamethasone; Bayer HealthCare) and oral doses of Baytril (enofloxacin; Bayer).

### Outline of the Experimental Design

**Figure 2:**
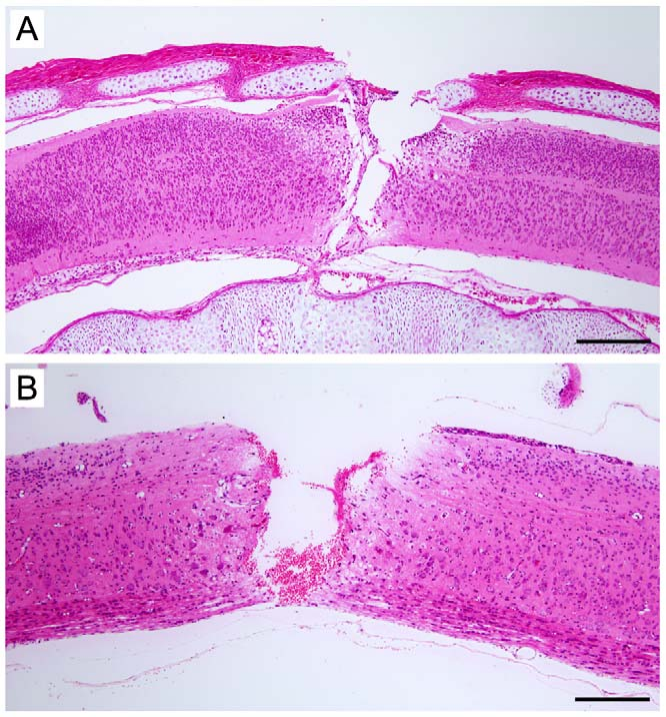
Sagittal H&E stained sections of the site of injury immediately following surgery. ***A:*** P7; ***B:*** P28. Orientation is rostral to the left, dorsal above. Scale bar = 200 µm.

Complete spinal cord transection was performed in the thoracic (T)10 region at one of two ages: postnatal day 7 (P7) or postnatal day 28 (P28). Gross morphological appearance of spinal cords at both ages immediately after surgery is illustrated in Fig. 2. Following surgery, injured pups were returned to the mother and allowed to grow in the usual environment until they were approximately 3 months old (young adults). The locomotor ability of these animals was assessed using a range of behavioural tests. Following these tests, retrograde axonal tracing was used to determine the degree of axonal growth through the injury site. Segments of the spinal cord containing the injury site (or equivalent area of control spinal cords) were fixed and used for histological staining (Haematoxylin & Eosin and Luxol Fast Blue).

A total of 40 animals were used in this study. P7-injured animals (n = 14), P28-injured animals (n = 10) and littermate controls (n = 16) were all assessed using a series of behavioural tests (BBB, grid test and swimming test, see below). A subset of these animals was also used for treadmill gait analysis (n = 5 for P7-injury group; n = 6 for P28-injury group; n = 4 for control animals). Seven to nine animals from each age group were used for retrograde labelling of supraspinal axons and an additional three from each group were designated for labelling of propriospinal neurons in the spinal cord. At the end of these experiments spinal cords from each group (n = 5 control, n = 9 P7-injury, n = 6 P28-injury) were processed for histological sectioning and staining. Each procedure is described in detail below.

#### Complete spinal transection at P7

Mothers were anaesthetised using inhaled isofluorane (5% in O_2_) and placed supine to expose the pouchless abdominal area to which the neonatal pups attach. The mother was maintained under constant isofluorane anaesthesia on a heated pad (25–28°C) for the duration of the operation, which never lasted more than 1 hour. Individual pups were additionally anaesthetised using a small tube filled with isofluorane-soaked cotton wool placed over the snout. A small sagittal incision was made in the skin overlying the lower-thoracic vertebrae (≈T10) and, using a fine ophthalmic blade (15° stab blade, Sharpoint), a laminectomy was performed to expose the spinal cord. Complete spinal transection was made using fine scissors (5 mm blade, Fine Science Tools). The wound was closed and sealed using surgical glue (Vetbond tissue adhesive, 3 M). When injuries were made at P7, all pups in a litter (6–7 pups) were injured. Controls for this age group were obtained from separate litters of pups, because these very young animals cannot be reliably marked without increasing the risk of cannibalism by the mother [7].

#### Complete spinal transection at P28

P28 pups were removed from their mother, anaesthetised individually using inhaled isofluorane via a facemask and maintained on a heated pad under continuous isofluorane. A sagittal incision was made in the dorsal skin over the lower thoracic vertebrae, and the musculature overlying the spinal column was incised to expose the vertebral spines. A laminectomy was performed at T10 and a complete cord transection was made using an ophthalmic blade. Throughout the procedure the spine was stabilised using a stereotaxic frame. Following transection the laminectomy site was closed and the wound sealed using surgical glue. All pups were returned to the mother after they had recovered from anaesthesia under a heat lamp for 1 hour. Injuries at P28 were usually made on half of the pups in a litter since at P28 their ears are easily marked. The remaining pups were anaesthetised as normal but remained uninjured and were used as controls.

#### Completeness of the spinal transaction

To confirm the effectiveness of the lesioning technique, some randomly selected operated pups at each age were exsanguinated under anaesthesia immediately after surgery and their cords were removed, fixed and sectioned for histology (see below). Spinal cord transection sites could be clearly distinguished by H&E staining (Fig. 2). Spinal transections performed at P7 were consistently found to be complete, as shown in Fig. 2A and as described previously [11], [18]. P28 transections (Fig. 2B) proved more variable with occasional tissue bridges being left intact. In the present study all of the P7-injured animals and only those P28-injured animals that were subsequently found to have complete transections were included in the analysis; this was defined by inspection at the time of removing the spinal cord and morphological examination of the site of injury after tissue preparation.

### Behavioural Studies

Comprehensive behavioural studies were performed on P7-injured, P28-injured and age-matched controls when the animals reached 90–100 days of age. These tests were only performed at a single time, approximately one month after weaning.

#### Open field locomotion

Open field locomotion was assessed using the Basso, Beattie, Bresnahan (BBB) Locomotor Rating Scale [12]. The BBB is a semi-quantitative 21-point scale designed to reflect locomotor recovery in rats with spinal contusions or complete transections [23]. However, the scale has been validated for another species of opossum, *Didelphis virginiana*, with complete spinal cord transections [8].

Opossums were encouraged to move about in a standard polycarbonate animal housing box (30 cm×40 cm) with a smooth non-slip floor. The animals were allowed to acclimatise in the box for 10 minutes prior to testing. Two trained examiners (BJW and JKC) observed each animal for 4 minutes and a score was assigned. The animals were encouraged into constant motion by gently tapping or scratching the sides of box. The observers were blinded as to the injury status of the animals. To encourage the animals' exploratory motion, all testing was performed under ambient light provided by a single low-wattage desk lamp fitted with a diffuser.

#### Gait Analysis

To further assess locomotor coordination animals were placed on a treadmill moving at a speed of 6 metres per minute (see Video S1) and digital video recordings were made. Footage of this treadmill locomotion was analysed frame-by-frame and the placement and lift-off of each limb was plotted for two periods of at least 4 complete step cycles. From these gait recordings the regularity index (RI) could be calculated. Regularity index is defined as the number of normal step sequence patterns (NSSPs) [24] expressed as a percentage of total paw placements: (RI = [(NSSP ×4)/number of paw placements]×100 [25], [26].

#### Grid test

Opossums were placed on a horizontal 1-metre ladder apparatus with metal rungs spaced one centimetre apart. Some rungs were randomly removed to create gaps in the ladder. Digital video recordings were made from two crossings of the grid and analysed later once blinded to the viewer. Every time the animal's foot fell below the level of the rungs, an error was recorded. The number of paw placement errors made during the crossings was expressed as footfalls per metre.

#### Swimming test

A glass tank measuring 1 metre long, 20 cm wide, 60 cm deep was used. An easily visible platform was placed at water level at one end of the tank. Opossums were placed first on the platform and allowed to become accustomed to it before they were placed in the water at the opposite end. Each animal was required to swim the length of the tank and climb onto the platform. Their swimming was recorded on digital video, which was later examined to see whether the animals were able to use their hindlimbs (evidence of supraspinal innervation).

#### Video processing

Videos were recorded using a Casio EX-F1 camera. Video analysis was performed using the QuickTime player.

### Morphological studies

#### Supraspinal axon tracing – single label protocol

Two to three weeks after behavioural testing was finished fluororuby (tetramethylrhodamine-labelled dextran amine, 10,000MW; Molecular Probes) was injected bilaterally into the spinal cord below the injury site. The intention of this experiment was to label any brainstem neurons whose axons had bridged the spinal lesion site. Comparable injections were made in control animals. All animals were anaesthetised under inhaled isofluorane and the spinal column was exposed. Using a sterilised dentistry drill (Foredom) fitted with a 0.7 mm stainless steel burr, holes were drilled bilaterally through the dorsal plate of the L2 vertebra to expose the spinal cord. The dura mater overlying the injection site was pierced using a fine glass pipette (70 µm outer diameter) and fluororuby (0.55 µl per injection; 25% w/v dissolved in 0.1 M Tris buffer with 2.5% (v/v) Triton X-100) was injected using gentle pressure into the tissue with a pulled glass micropipette attached to a PVC tube. The area was immediately washed with sterile saline (0.9% NaCl), Gelfoam was placed over the dura and the wound was sealed using tissue glue, as described above. The animal was returned to its box for 5 days before further study.

#### Propriospinal axon tracing – double label protocol

In some animals a double labelling protocol was employed. A unilateral injection of fluororuby was made below the injury site, as described above, and at the same time a unilateral injection of Oregon green–labelled dextran amine (10,000 MW, Molecular Probes; 25% w/v dissolved in 0.1 M tris buffer with 2.5% (v/v) Triton X-100) was made above the injury site (T7/T8), using the same technique. This protocol was used to investigate the possibility of propriospinal neurons crossing the injury site.

#### Tissue Fixation

Five days following injection of fluorescent dextran amines opossums were terminally anaesthetised using i.p. injections of Urethane and perfused transcardially with cold heparinised saline (0.9% saline with 5 U/ml heparin) followed by 10 minutes perfusion of cold paraformaldehyde (4% paraformaldehyde in phosphate buffered saline, PBS) at a rate equal to 75% of total blood volume per minute. Total blood volume was estimated as 10% of body weight. The spinal cord and brain were removed and post-fixed in paraformaldehyde for 24 h. Brains were embedded in 4% agar and stored under PBS at 4°C until further processing. A 10 mm segment of the spinal cord containing the injury site (or an equivalent segment in control animals) was removed and either post-fixed in Bouin's fixative overnight for histological processing or embedded in agar.

#### Axon tracing analysis

Brains and spinal cords embedded in agar were sectioned at 100 µm using a vibrating microtome (Leica VT1000S) and mounted on glass slides in fluorescent mounting medium (DAKO). Brain tissue was sectioned in the coronal plane; spinal cord tissue was sectioned in the transverse plane. Sections were viewed under a microscope (Olympus BX50 fitted with a DP70 camera) under appropriate Olympus fluorescent filters. Mono- and Dichrotic mirror/filter combinations specific for the wavelength of each fluorophore were used to visualise final mono- and bi-fluorescent images. Sections through the injection site were examined to establish that the injections were successful. For the single label protocol any animal that showed only unilateral dye distribution was considered an unsuccessful injection and was removed from the analysis. For the double label protocol unilaterality of the dye was confirmed before further tracing analysis was performed.

Every coronal section through the brainstem was examined and all fluorescent neurons were counted and assigned to individual nuclei (as described in detail in [11]). Brainstem nuclei were defined with reference to Oswald-Cruz and Rocha-Miranda's *Didelphis marsupialis* brain atlas [27], which has previously been used for several species of opossum, including *Monodelphis domestica*, [8], [9], [11], [28], and a *Mondelphis domestica* brain atlas developed in the laboratory [29].

#### Histological processing and staining

Cords containing the injury site (and equivalent areas of control spinal cords) were processed for paraffin embedding and sectioned in the transverse plane at 5 µm with a microtome. Ten sections were mounted on each slide and every tenth slide was stained with H&E for general morphological examination, using standard methods. Slides adjacent to these were stained for myelin using the Luxol® Fast Blue (LFB) method, as described previously [30]. Briefly, tissue was dewaxed and cleared in 100% followed by 95% ethanol. Slides were stained overnight at 60°C in Luxol® Fast Blue MBSN (0.1 g; Sigma Aldrich) dissolved in 10% acetic acid and ethanol. Sections were rinsed in 95% ethanol and then differentiated in 0.05% lithium chloride solution followed by 70% ethanol. Tissue was then dehydrated through graded ethanol and histolene (Fronine) and mounted with DPX mounting medium (Fronine).

#### Morphometric analysis

Sections stained with Luxol Fast Blue were used for quantitative analysis of spinal cord morphology. All images were captured using an Olympus DP-70 camera attached to a compound microscope (Olympus BX-50) using identical camera, lens and exposure settings. Image analysis was performed using ImagePro Plus software (version 4.5.1.22). One section from every stained slide (every 500 µm along the 10 mm segment of spinal cord) was used to analyse the cross-sectional tissue area. Luxol Fast Blue–positive material was automatically detected and measured using ImagePro Plus software. For analysis purposes these data were aligned so that the centre of each cord's injury site overlapped precisely with the others from each group to give a profile of lesion size and myelin content surrounding the injury centre. Images throughout the length of spinal cord centered on the injury site were overlayed using Adobe Illustrator (version 10.0.3) to reconstruct the cord and demonstrate its gross morphology.

#### Correlating morphological measurements and behavioural scores

Morphological measurements and functional scores were plotted against each other so that relationship between these variables could be assessed. The three morphological measures chosen for correlation were (i) the number of back-labelled brainstem neurons, (ii) the volume of spinal cord tissue immediately surrounding the injury site (6 mm) and (iii) cross-sectional area of the spinal cord at the centre of the injury site. These were plotted against either BBB score or the number of grid test foot placement errors, obtained as described above.

#### Statistical analysis

All data are presented as mean ± standard error of the mean (SEM). All data were analysed using One-way Analysis of Variance (ANOVA) and individual differences were determined using a Bonferroni post-test, except for the counts of labelled neurons in individual brainstem nuclei, where Student's t-test was used to determine statistical differences between the two groups. Correlations were performed using GraphPad Prism software. *P*<0.05 was considered statistically significant.

## Results

*Monodelphis* pups at either P7 or P28 underwent a complete spinal transection at T10 and were left until adulthood along with age-matched un-operated animals that were used as controls. All animals were tested using several behavioural methods (see Methods) followed by axonal tracing experiments; subsequently, spinal cord tissue was collected for morphological analyses.

### Gross morphology

**Figure 3:**
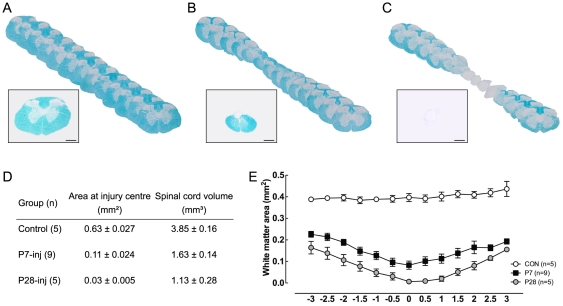
Reconstruction of spinal cord gross morphology for adult animals. ***A:*** Control; ***B:*** P7-injured; ***C:*** P28-injured spinal cord. Sections (500 µm apart) were stained for Myelin (Luxol Fast Blue stain) and overlayed using Adobe Illustrator. Inset images are representative sections through the centre of the injury site (or equivalent spinal level in control cords). Scale bar is 200 µm. ***D*** *:* Average tissue cross-sectional area at injury centre and spinal cord volume. **E:** Myelin-positive white matter area in serial sections of 6 mm segment from spinal cord incorporating the injury site. Numbers on the x-axis refer to distance from the centre of injury (0) in mm.

Myelin staining (Luxol Fast Blue) was used to obtain a general morphological overview of the injury area after the opossums had grown to adulthood. Images of every 10^th^ section throughout the 10 mm length of cord centered on the site of injury were overlaid so that gross cord architecture could be viewed. Representative overlays are shown in Fig. 3A–C with higher magnification images provided as insets in these figures. Histological examination of spinal cords taken from adult animals that were injured at P7 revealed that a dense tissue bridge, which stained positive for Luxol Fast Blue, had spanned the injury site, demonstrating a re-growth and reconnection of the two ends of the previously severed spinal cord (Fig. 3B). However, this re-established spinal cord tissue was narrower at the lesion centre (0.11±0.02 mm^2^, n = 9) compared with age-matched control spinal cords (Fig. 3A, 0.63±0.03 mm^2^, n = 5, *P*<0.001). Sections obtained from the spinal cords of animals injured at P28, on the other hand, showed a very different tissue structure in the site of injury (Fig. 3C). A loose halo of unidentifiable tissue, which did not show a positive reaction with Luxol Fast Blue, connected the two ends of the severed spinal cord and surrounded a cyst-like cavity that spanned the injury site. Morphometric analysis of cord tissue cross-sectional area at the centre of the injury and tissue volume in the injury site are tabulated in Fig. 3D. The profile of Luxol Fast Blue positive material in the spinal cord tissue surrounding the injury site is shown in Fig. 3E. In P7-injured animals myelin was detectable in the injury centre and although the normal organisation of the cord into white and grey matter was disturbed, it was still identifiable. This was no longer the case in P28-injured animals as no myelin was detected in the injury site of these animals. Injury at P7 or P28 resulted in similar lengths of myelin deficiency in the cord but the actual loss is greater in the P28-injured animals.

#### Retrograde tracing analysis

**Figure 4:**
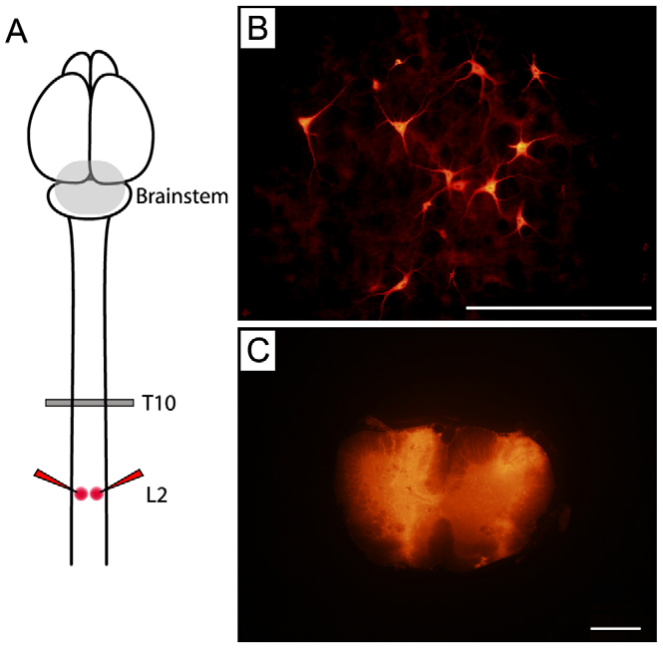
Retrograde labelling of brainstem neurons. ***A:*** Schematic diagram of the injection protocol. Fluororuby was injected at L2. The label was transported back to the cell bodies of those brainstem neurons that had projected axons across the injury site at T10. The grey area in the brainstem represents the area of analysis; ***B:*** Representative image of brainstem neurons labelled in the gigantocellular reticular nucleus in the medulla; ***C:*** Representative bilateral injection into the L2 segment. Scale bars = 200 µm.

In order to establish which, if any, brainstem neurons had projected processes across and beyond the site of injury, adult animals that had been operated at P7 or P28, together with age-matched controls, were injected with a fluorescently tagged dextran amine (fluororuby) below the level of transection (Fig. 4A). Five days later the animals were killed, perfuse-fixed and agar-embedded brainstems were sectioned using a vibrating microtome at 100 µm. Each section was viewed under a fluorescent microscope and all fluorescent neurons were counted and mapped to individual brainstem nuclei. Fig. 4 shows a schematic diagram of this labelling protocol (Fig. 4A), fluorescent neuronal labelling in the brainstem (Fig. 4B) and representative images of a successful injection site (Fig. 4C). Animals found to have unsuccessful injections were removed from the study (2 animals from P7-injured group, 1 from P28-injured group).

**Figure 5:**
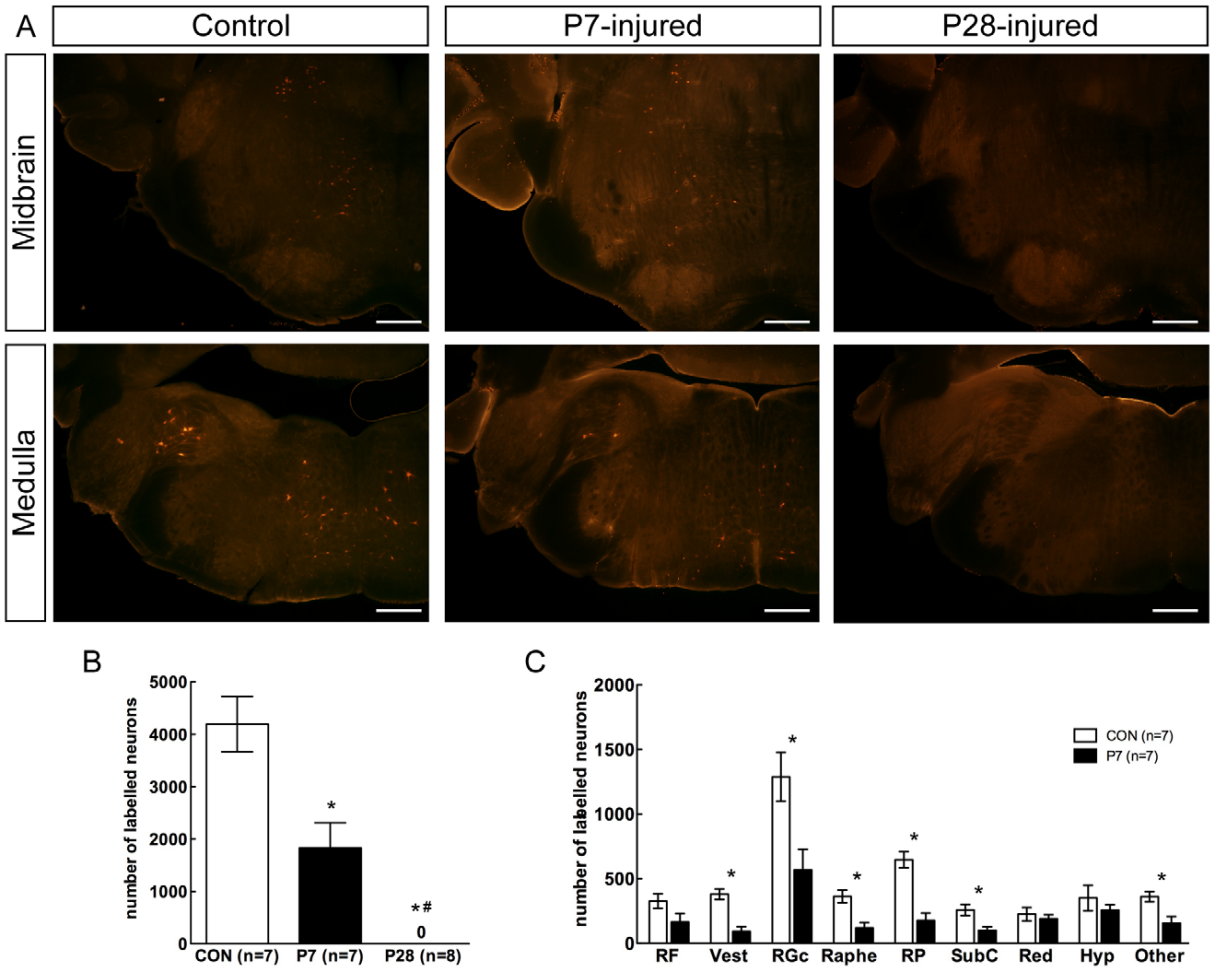
Labelling of brainstem neurons. ***A***: Fluorescently labelled neurons in the midbrain (upper panels) and rostral medulla (lower panels) of control, P7-injured and P28-injured animals using 10× objective under dichrotic mirror/filter combination specific for the wavelength of each fluorophore. Scale bar = 500 µm; ***B:*** Number of fluorescently labelled brainstem neurons in control (CON) and injured opossums; ***C:*** Number of labelled neurons in individual nuclei in the brainstem of control and P7-injured opossums. Note that there were no labelled neurons in the brainstems of P28-injured animals. Data are mean±sem. * *P*<0.05 vs control. # *P*<0.05 vs P7-injured. Abbreviations for brainstem nuclei: RF: reticular formation; Vest: vestibular nuclei; RGc: gigantocelluar reticular nucleus; Raphe nucleus; RP: reticular pontine; SubC: sub-coeruleus; Red nucleus; Hyp: hypothalamic nuclei; Other: spinal trigeminal nucleus, nucleus ambiguus, Edinger-Westphal nucleus and the interstitial nucleus of the medial longitudinal fasciculus.

Brightly fluorescent neuronal cell bodies could be seen under low magnification (10×) at all levels of the brainstem in control and P7-injured animals, whereas no labelled cell bodies could be found in any areas of the brainstem of P28-injured animals (see Fig. 5A for representative examples). In brainstems of P7-injured animals, significantly fewer labelled neurons were detected compared with controls (Fig. 5B). In control animals the major brainstem nuclei that contained labelled neurons were the gigantocellular reticular, the reticular pontine, the vestibular (medial and lateral), the red, the raphe, the hypothalamic, sub coeruleus and the dorsal and ventral medullary reticular matter. These typically accounted for over 80% of total counts in control animals. The remaining nuclei were classified as ‘other’, and included spinal trigeminal nucleus, nucleus ambiguus, Edinger-Westphal nucleus and the interstitial nucleus of the medial longitudinal fasciculus. No labelling was found in cortical regions of the brains of any animal in this study, in accordance with previous observations for opossums [8], [11]. In the brainstems from P7-injured animals fluorescently labelled neurons were found in all of the same brainstem nuclei as controls, although in lower numbers except in the reticular formation, red nucleus and the hypothalamic nuclei, which showed numbers of labelled neurons that were similar to controls. The distribution of labelled neurons in control and P7-injured opossum brainstems is shown in Fig. 5C.

### Behavioural studies

**Figure 6:**
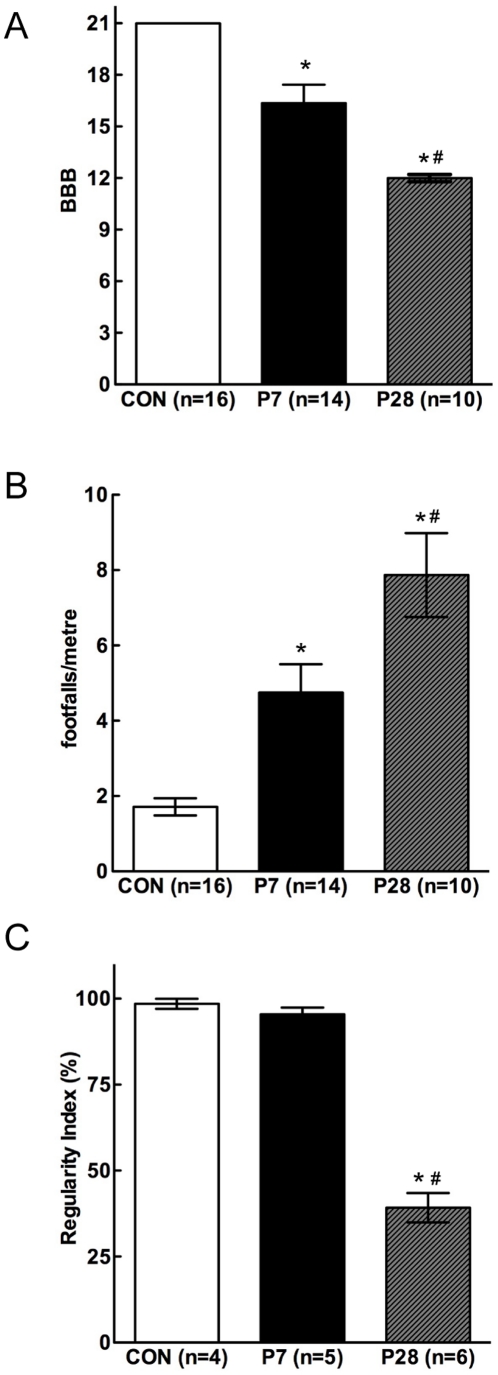
Behavioural analysis of adult opossums injured at P7 or P28 compared with control animals. ***A:*** BBB locomotor analysis; ***B:*** Grid test foot placement errors; ***C:*** Regularity index. All data are mean±s.e.m. * *P*<0.05 vs control. # *P*<0.05 vs P7-injured.

Motor function was assessed using behavioural tests aimed at measuring a variety of behavioural abilities and included open field locomotion, interlimb coordination, and voluntary control of hindlimb movements. The results from these tests are shown in Fig. 6.

#### Open field test

In order to assess the use of hindlimbs in the open field, the 21-point Basso, Beattie and Bresnahan (BBB) Locomotor Rating Scale was used (Fig. 6A). Opossums injured at P7 displayed a clear use of the hindlimbs in this setting (BBB 16.4±1.1). All animals were able to support their weight fully and more than half achieved consistent forelimb–hindlimb (FL–HL) coordination. Of the 14 P7-injured opossums used for the BBB analysis 6 did not achieve consistent FL–HL coordination and thus scored lower on the rating scale. The remaining 8 animals walked well but common deficits remained in their locomotor abilities; in particular, trunk instability and rotational errors of contact and lift off of the hind paws. These deficits led to the P7-injured group scoring significantly lower (16.4±1.1) than controls (21±0; *P*<0.001) on the BBB scale (Fig. 6A).

Opossums injured at P28 were also able to walk surprisingly well, considering their anatomical deficits and scored 12.0±0.21 on the BBB scale (Fig. 6A). Of the ten P28-injured opossums used for this analysis all could take plantar steps with apparent full weight support, though none achieved consistent FL–HL coordination. Thus, they were all scored between 11 and 13 on the scale, depending on the degree of coordination that was observed; these scores were significantly lower on the BBB scale than for both control (*P*<0.001) and P7-injured groups (*P*<0.001).

Several other non-BBB-assessed behavioural parameters were observed in the open field. Normal opossums commonly rear up onto the sides of their box. Most P7-injured animals could perform this task spontaneously at some point during the acclimatisation/assessment period, but this behaviour was never observed for P28-injured animals. Grooming behaviour normally occurs in opossums by crouching on the hindlimbs and using both front paws to groom the face. P7-injured animals were usually able to perform this task, but P28-injured animals instead groomed with one front paw at a time, presumably because they were unable to balance proficiently on their hindlimbs (not illustrated).

#### Grid test

In the grid test opossums were assessed as they walked along a narrow ladder with rungs randomly removed to expose 2 cm gaps in the grid. Errors in hindlimb placement were counted. This test requires careful hindpaw placement and substantial motor control [31]. Results are shown in Fig. 6B. P7-injured animals made significantly more errors than controls (4.75±0.76 versus 1.71±0.23 errors per metre, *P*<0.01). P28-injured animals made more errors (7.87±1.12 errors per metre) than either control (*P*<0.001) or P7-injured (*P*<0.01) groups.

#### Treadmill test

To further assess FL–HL coordination some animals (n = 4–6 per group) walked on a treadmill running at 6 metres/minute and all paw placements and lift-offs were plotted. Interlimb coordination (the Regularity Index) was measured from these plots. Regularity Index (RI) is defined as the number of normal step sequence patterns (NSSP; any four sequential steps from all four limbs placed in any order) expressed as a percentage of total paw placements. Representative gait plots and video of opossums performing this task can be found in Fig. S1 and Video S1. In this test both control (RI 98.5±1.5%) and P7-injured (RI 95.5±1.9%) opossums walked with highly regular paw placements, whereas P28-injured opossums fared significantly worse (RI 39.2±4.3%, *P*<0.001 compared with control). Results are shown in Fig. 6C.

#### Swimming test

**Figure 7:**
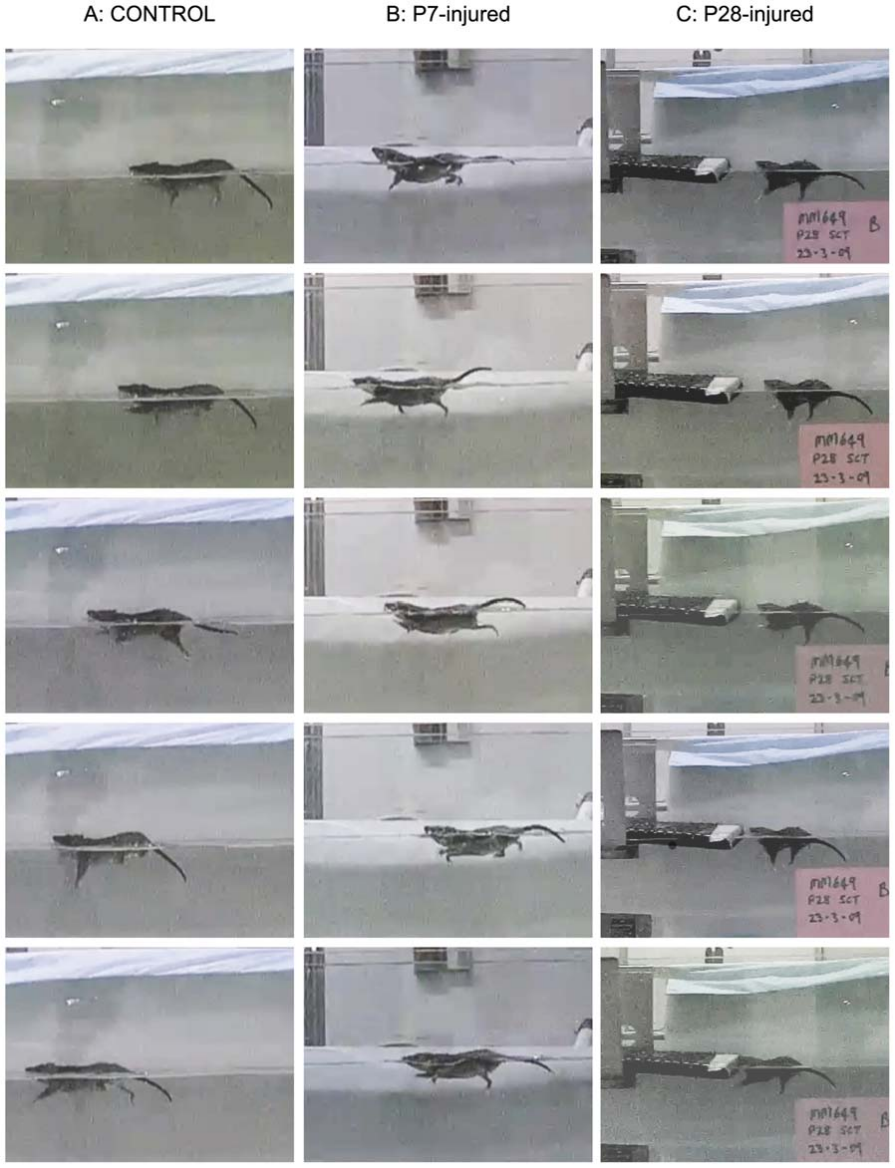
Video stills from the swimming test. These representative still images from swimming test videos show forelimb and hindlimb movements of adult animals during swimming. ***A***: control opossum, ***B***: P7-injured opossum and ***C***: P28-injured animal. Note that both control and P7-injured animals used fore- and hindlimbs but P28-injured opossums were only able to use forelimbs.

Swimming places the animals in an environment of low peripheral sensory feedback [7]. Examples of control and transected animals performing this test are shown in Fig. 7A–C. Control opossums swim strongly and, unlike rats, use all four limbs in the swimming stroke (Fig. 7A). P7-injured opossums were also able to utilise all four limbs when swimming, albeit in an apparently less coordinated manner (Fig. 7B). P28-injured animals, on the other hand, swam with complete dependence on the forelimbs, and were unable to move their hindlimbs (Fig. 7C). Some rocking of the trunk was observed in these animals, but body position in the water appeared otherwise normal. When the P28-injured animals climbed out of the water onto the platform, they were once again able to use their hindlimbs as soon as their feet came in contact with the platform, as observed in the locomotor testing above. Representative video footage of this test can be found in Video S2.

#### Correlation of morphological measures and behavioural recovery

**Figure 8:**
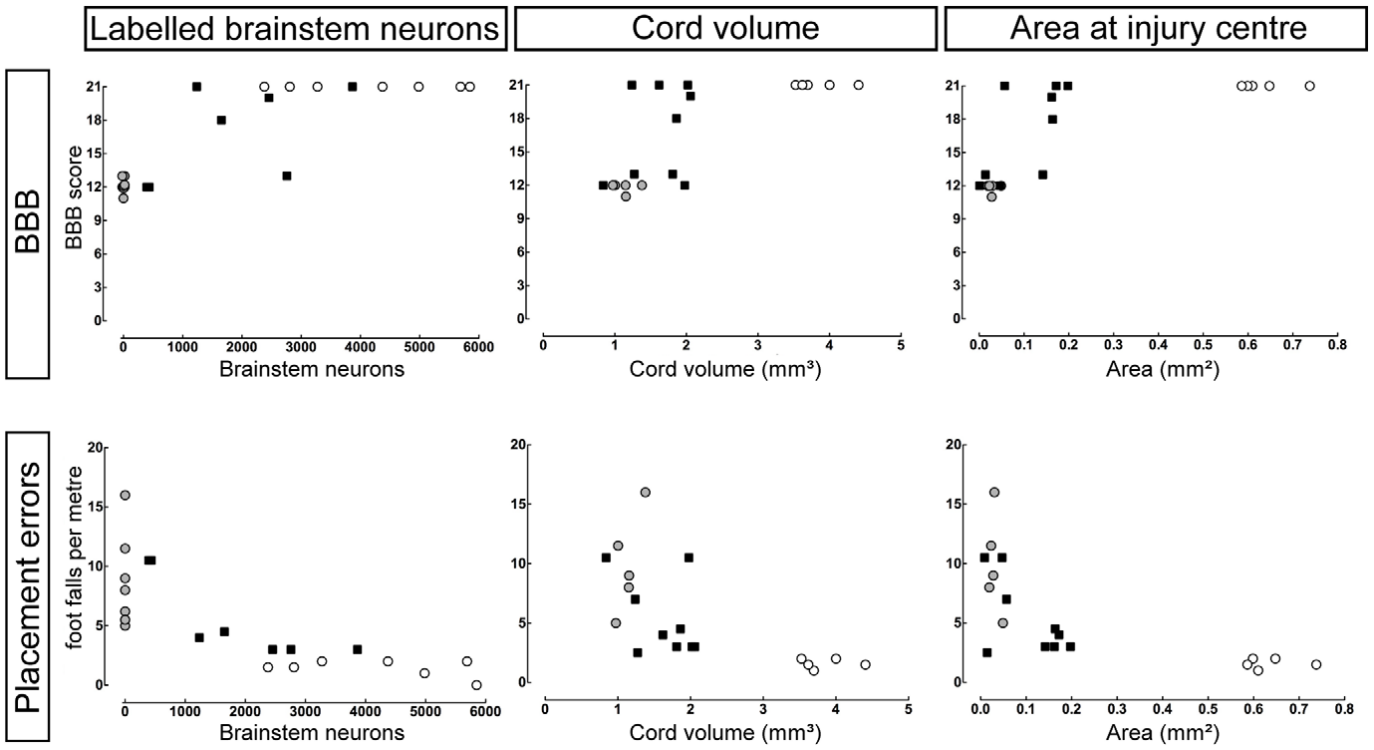
Correlations of structure and function. Independent variables (numbers of labelled brainstem neurons, spinal cord volume and cross-sectional tissue in injury centre) are listed across the top; dependent variables (BBB locomotor score and grid test foot Placement errors) are listed on the left. Controls (open circles), P7-injured (black circles) and P28-injured animals (grey circles) are plotted in the grid of graphs. Correlations were calculated for P7-injured group only. None was strongly correlated except grid test foot Placement errors vs Labelled brainstem neurons (*r* ^2^ = 0.68, *P*<0.05; lower left).

The relationship between morphological repair and behavioural recovery following injury was investigated. Two behavioural measurements (BBB score and grid test foot placement errors) were correlated with three morphological measures (numbers of back-labelled brainstem neurons, volume of spinal cord tissue and spinal cord cross-sectional area at the centre of the injury site) and lines of best fit were assigned using Graphpad Prism software. The results are shown in Fig. 8. Animals from all three groups (control, P7-injured and P28-injured) have been included, although only the P7-injury group has been used for making the correlations, because that was the only group that displayed a wide range of morphological repair allowing meaningful statistical analysis. As can be seen from Fig. 8 the only two measures that were significantly, though only weakly, correlated were foot placement errors (grid test) and numbers of back-labelled brainstem neurons (*r* ^2^ = 0.69, *P*<0.05). All other correlations had *r* ^2^ values below 0.45 (Fig. 8).

#### Propriospinal labeling

**Figure 9:**
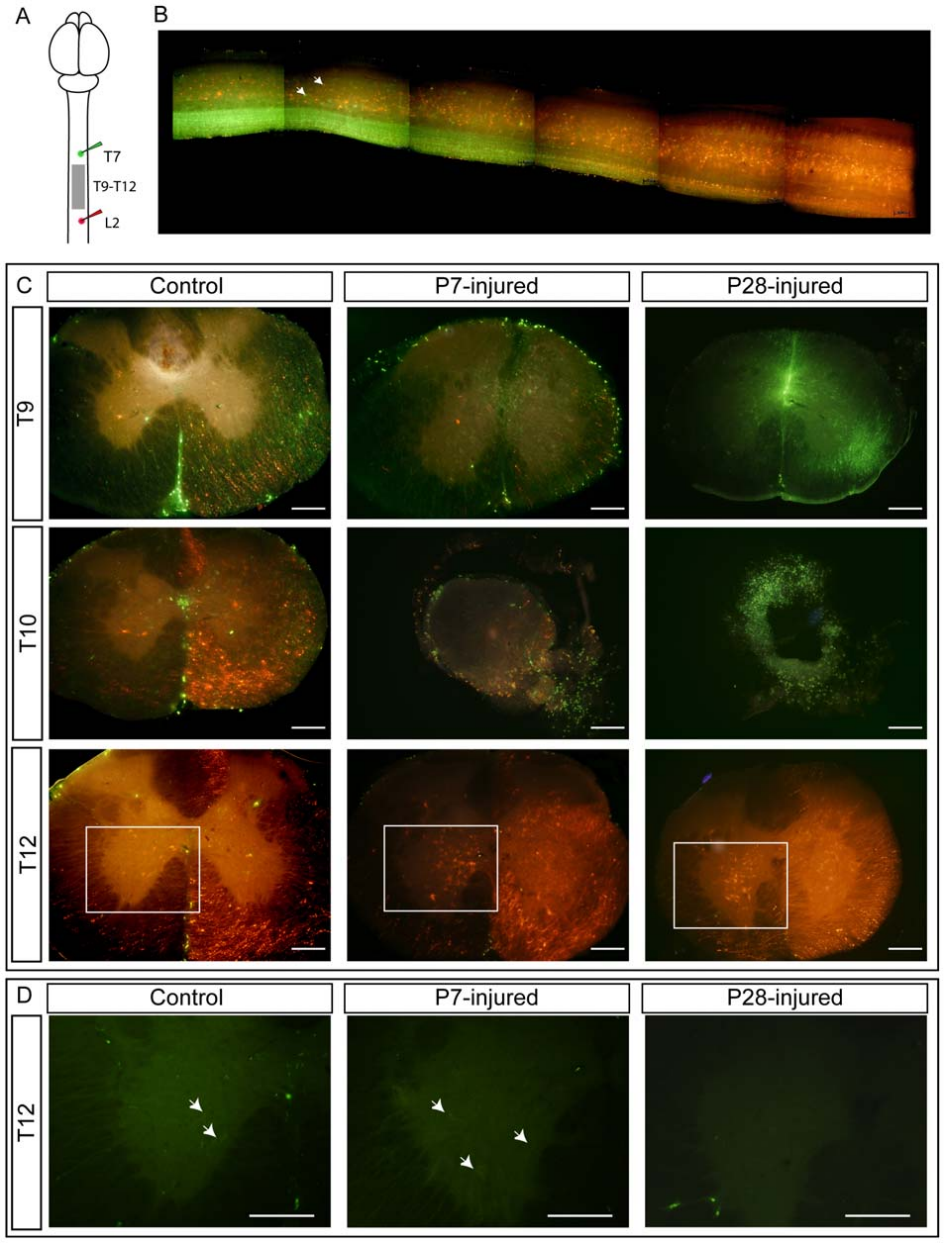
Fluorescent labelling of propriospinal neurons. ***A:*** Schematic diagram of labelling protocol. Oregon green was injected into T7 and Fluororuby was injected into L2. The area between the injection sites (T9–T12; grey box) was examined for labelled cell bodies. ***B:*** Parasagittal section through the T9–T12 region of a control spinal cord showing fluorescently labelled cell bodies in the spinal grey matter (arrows). Note the presence of both fluorescent labels along the whole cord segment. ***C:*** Images under a dichrotic filter of transverse sections of spinal cord segment from control, P7-injured and P28-injured animals showing the labelling of propriospinal neuronal cell bodies below the injury site (lower panels), at the centre of the injury site (middle panels), and above the injury site (upper panels). ***D:*** Representative higher magnification images (of boxed areas in ***C***) taken under only a monochrotic filter detecting green Oregon green positive neurons in the spinal cord below the injury site. This was done because the green fluorescence signal is much weaker than red under dichrotic filter, especially close to the Fluororuby injection site. Arrows in D indicate Oregon green positive neurons. Scale bar = 200 µm.

To determine whether the axons of any non-supraspinal neurons crossed the injury site a double labelling protocol was used (Fig. 9). Injections of different fluorescently labelled dextran amines were made above (T7/8; Oregon green) and below (L1/2; Fluororuby) the injury site. The spinal cord was then sectioned in transverse plane at 100 µm and the spinal grey matter between the injection sites (T8–T12) was examined for labelled cell bodies, with specific regard to cells that were labelled with the fluorophore that was injected into the cord on the other side of the injury (Fig. 9A). To establish the distance to which propriospinal neurons could be labelled, one control spinal cord was sectioned in the sagittal plane. Fig. 9B shows a 10 mm reconstructed sagittal image through a control spinal cord exhibiting abundant labelling of propriospinal interneuronal cell bodies. These cell bodies were labelled with either Oregon green or fluororuby, never both, and populations containing each label were distributed along the length of the spinal cord. This confirmed that the propriospinal neurons found within the opossum spinal cord are capable of projecting axons for sufficient distances for it to be possible to determine if they had crossed the injury site. This is confirmed in transverse sections shown in Fig. 9C. As can be seen in control cords, (Fig. 9C left column), separate cell populations were labelled with only one fluorophore, in the grey matter on both sides of the spinal cord and at all spinal levels examined (T9–T12). The spinal cords of opossums injured at P7 also contained distinct populations of labelled neurons labelled with each fluorophore (Fig. 9C, centre column). Fewer fluororuby-positive cell bodies were present in areas rostral to the injury site than in controls; however, their presence suggests that propriospinal neurons, like supraspinal neurons described above, are able to project processes across the injury site. In contrast, in P28-injured animals no fluororuby-positive cell bodies rostral to the injury site or Oregon green labelled neurons caudal to the injury site could be detected (Fig. 9C right column). Fig. 9D shows higher magnification images from the T12 segment under only the green-detecting filter to show the presence of Oregon green–labelled neurons in the cords of control and P7-injured animals below the injury site, and the absence of these in the cords of P28-injured animals. The use of the filter specific for the green fluorophore only for these is due to the fact that the bi-fluorescent filter used in Fig. 9C detects Oregon green fluorescence weakly, especially when it is present near an area of high red-fluorescent intensity such as the fluororuby injection site. Thus, cords of P28-injured animals showed no evidence of propriospinal projections crossing the site of the spinal injury.

## Discussion

This study has shown in *Monodelphis domestica* when examined as adults there is no detectable growth of axons across a complete spinal cord transection made at P28, when the animals are examined after growing to adulthood. Thus, there was no backlabelling of brainstem neurons when fluororuby was injected caudal to the lesion (Fig. 5) and local injections of tracer above and below the lesion did not result in any labelling of axons originating from intraspinal neurons crossing the lesion.

This study also confirmed, as shown previously [11] that a complete transection at P7 was followed by substantial growth of axons across the lesion and that the neurons of origin of at least some of these axons could be backlabelled by an injection of fluororuby caudal to the lesion. The total number of neurons labelled in these P7-injured animals was less than in controls but the labelling was present in the same brainstem nuclei and in similar proportions to those in the control animals.

Behavioural studies showed that there was a marked difference in the behaviour of animals with cords transected at P7 or at P28 when tested as adults. As previously reported by Saunders et al. [7] in *Monodelphis domestica* and by Wang et al. [8] in *Didelphis virginiana*, animals transected in the first week of life developed a behavioural performance that approached normal. The injuries in our earlier study [7] in *Monodelphis* were made either by crushing the cord with fine foreceps or by complete transection, as described here, albeit at a different spinal level and all at P7. The morphological repair following crush injury was more complete than following a lesion made with a microknife, though the behavioural responses were comparable. The completely transected animals in the previous study performed locomotor and swimming tasks to a similar level as the P7-injured animals described here.

In the present study the P7-transected animals had BBB scores of 16.4±1.1 compared with a normal control score of 21. However, the range of BBB scores indicated that there was a variation in the degree of recovery, with some animals scoring as high as 21 (Fig. 8). This will be discussed further in relation to the back-labelling studies and the other behavioural tests, below.

The most notable finding in the present study was the extent of locomotor behaviour exhibited by the P28-transected opossums when tested as adults. As indicated above, these animals showed no evidence of axonal growth across the lesion yet they exhibited body weight bearing locomotion, which at times appeared to have some degree of FL–HL coordination since the BBB score was 12.0±0.21 (a level designated in the BBB rating scale as “frequent to consistent weight-supporting plantar steps and frequent FL–HL coordination” [12]). However, gait analysis (see Fig. 6C) showed that the Regularity Index (a measure of inter-limb coordination) in the P28-transected opossums was measurable (39.2±4.3%) but much less than in either P7-transected animals (95.5±1.9%) or unoperated controls (98.5±1.5%). This was because about one third of the steps taken whilst running on the treadmill test appeared to be coordinated between fore- and hindlimbs, presumably as a matter of chance. This probably explains BBB scores indicating some degree of FL–HL coordination. Also, because *Monodelphis* tend to run when placed in an open field environment, it is more difficult to assess FL–HL coordination visually. This emphasises an important observer-dependent limitation of the BBB scale (even in rats which tend to walk rather than run) and the need for more objective measures of locomotion such as treadmill-based analyses.

Courtine et al. [32] used an ingenious combination of spatially and temporally separated lateral spinal hemisections combined with labelling of propriospinal neurons to show that much of the spontaneous recovery from spinal cord injury in adult mice could be explained by reorganisation of descending and propriospinal connections without regeneration or maintenance of direct projections from the brainstem. They then speculated that the resulting recovery of voluntary hindlimb locomotion was due to supraspinal signals being relayed through these novel circuits to the relevant motor areas below the injury. In our study, P28-injured animals achieved recovery of hindlimb stepping even in the absence of any these propriospinal fibres crossing the injury site (Fig. 9). Thus, the locomotion observed here was not supraspinally mediated, directly or indirectly. This interpretation is supported by the functional finding that in the swimming test P28-injured animals could not use their hindlimbs; presumably because in this environment their hindlimb spinal rhythm generator was not receiving sufficient sensory input to trigger locomotor-like actions. In contrast, as soon as the animals climbed out of the swimming tank onto the exit platform they were again able to use their hindlimbs (see Video S2).

**Figure 10:**
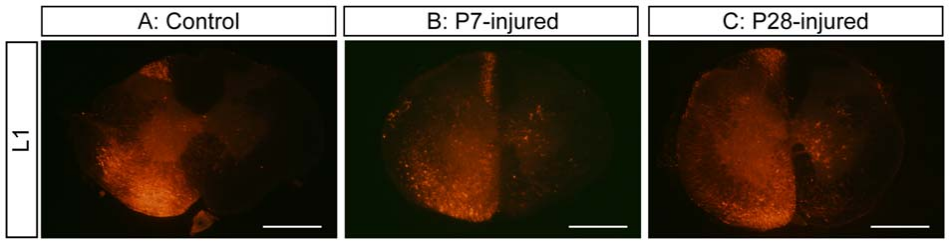
Fluorescent labelling of propriospinal neurons in the lumbar spinal cord. Fluororuby was injected into the L2 segment and the lumbar spinal cord was sectioned and examined for labelled propriospinal nuclei. Representative images from the L1 segment are shown here from Control (A), P7-injured (B) and P28-injured (C) opossum spinal cords. Note the contralateral labelling in the P7 and P28 transected spinal cords compared to control. This labelling is more marked in the P28 injured spinal cord, with both neuronal cell bodies in grey matter and axons in white matter being labelled. All images are taken under dicrotic filter. Scale bar = 500 µm.

It seems likely then that the locomotion observed in P28-injured animals in this study is entirely generated within the lower spinal cord, caudal to the injury site. Whether reorganisation of the intraspinal networks in this region is contributing to this recovery or whether adaptation of existing connections occurs is not yet known. From the labelling experiments already performed, we have observed a difference in the number and distribution of fluorescently labelled neurons in the lower spinal cord, below the level of the injury site (Fig. 9C lower panels). Although in this study this observation is restricted to the lowest segment of spinal cord examined (T12) and no formal counting has been performed, there seems to be a dramatic increase in the number of labelled neurons in the lower spinal cords of both P7-injured and P28-injured opossums compared with controls. This may well be an indication that reorganisation of the circuitry underlying locomotion is important for function. Preliminary images of fluorescent labelling in the lumbar spinal cord are shown in Fig. 10. These images suggest that, compared with control (Fig. 10A), the neuronal networks in the lumbar spinal cord are modified following injury at either P7 (Fig. 10B) or P28 (Fig. 10C).

In contrast to the P28-injuries, P7-injured animals were able to regrow propriospinal axons across their injury sites. They were also able to use their hindlimbs when swimming, suggesting that, at some level, supraspinal control had been re-established. It is not possible to say though, from the present study, what role the propriospinal axons that crossed the injury site play in either the locomotor responses or the swimming abilities of these animals. The lack of correlation between any functional improvements and labelling in the brainstem of these animals may provide some insight of the important role that these propriospinal neurons play.

Wang et al. [8] showed that in *Didelphis* with spinal cords transected in the neonatal period and re-transected when the animals were adult showed significantly higher BBB scores than adults transected for the first time. However, the main effect observed was an immediate and prolonged loss of hindlimb function, indicating that transected axons which had grown across the lesion were largely responsible for the substantial level of function in these animals prior to re-transection. The authors considered that the residual function detected once the animals had recovered from the surgery was probably due to both encephalisation of locomotion and progressive segmental inhibition, as part of normal development, although they pointed out that more research is needed [8]. We have carried out some preliminary studies with the aim of comparing the behaviour of adult animals with spinal cord transection to animals operated at P7 or P28 and re-transected as adults. Unfortunately these experiments had to be terminated before two weeks post-injury because of substantial autophagia of the hindlimbs.

### Recovery and development of function associated with axon growth across the lesion

The only unequivocal evidence of substantial spontaneous recovery of function from a complete spinal cord transection that is associated with axon growth across the lesion, at least part of which is from supraspinal connections, comes from the studies in marsupial species that are born at a much earlier stage of cord development than rats and cats [6]–[9]. The combination of backlabelling and multiple behavioural studies presented here allows some examination of the possible relation between the number of supraspinal neurons contributing to axon growth across the lesion and the level of behaviour that could be measured. Other measures of structural repair that might be expected to correlate with level of behaviour are estimates of the size of neural material (cross-sectional area or volume of myelin) of the spinal cord at the site of the lesion. Only foot placement errors (grid test) and labelled brainstem neurons showed a weak correlation in P7-injured animals (*r* ^2^ = 0.69, *P*<0.05). This apparent lack of correlation between structural repair and behaviour might be due to a contribution by non-myelinated axons growing across the lesion, the contribution of propriospinal neurons crossing the injury site (as discussed above), or more likely it may be due to many of the axons crossing the lesion but failing to make any or appropriate synaptic connections. Wang et al. [8] injected fluororuby into brainstem nuclei of neonatally transected and control adult *Didelphis*, using anterograde transport of the tracer to determine the distribution of neural connections caudal to the lesion. There was a similar location of marker in controls and operated animals but less profuse in the latter. This suggests that the axons crossing the lesion were able to reach appropriate target regions but does not establish how many made functional connections.

Fry et al. [11] estimated that about 50% of axons severed by transection at P7 regenerated but, because of continued growth of new axons into the spinal cord, by adulthood the proportion of regenerated axons in the total number of axons crossing the lesion site was about 5%. It is not at present possible to say what contribution to recovery of function these regenerated axons made; it is also not clear whether axons involved in functional recovery were making normal connections or if the animals learned to use aberrant connections that had been made, although the findings of Wang et al. [8] (above) suggest that the former is more likely. Nevertheless, an important conclusion which can be drawn from the present study is that only a proportion of the axons normally present at the level of injury appear to be required to cross the lesion and make functionally useable connections in order to establish near normal locomotor function. As observed in the P7-injured animal numbers of backlabelled neurons were about 45% of control numbers (Fig. 5); however, what is actually required for the level of function demonstrated may well be much less than this since it is likely that not all of the axons crossing the injury site made functionally effective connections. Estimates of morphological repair from myelin staining showed that both the cross-sectional area at the centre of the lesion and the volume of spinal cord tissue surrounding the lesion in operated animals were appreciably less than in controls (17% and 42%, respectively, see Fig. 3). Yet these animals displayed substantially normal overground locomotion and a high degree of forelimb–hindlimb coordination.

### Conclusion

A significant advantage of neonatal marsupial species like *Monodelphis* for studies of recovery after complete spinal cord transection is that, as shown in this study, it is possible to examine animals with cords transected at an age when recovery is associated with unequivocal growth of axons across the lesion and into the segment of the cord caudal to the lesion, compared with animals transected at a later stage when no axon growth occurs but there is still substantial recovery of locomotor function. It has not previously been possible to make this distinction as clearly in neonates of eutherian laboratory species such as cats and rodents, probably because they are already at a later stage of CNS development than the postnatal (marsupial) opossums used in this study. That the P28-transected opossums showed clear cut evidence of significant locomotor performance in the apparent absence of either supraspinal or local axonal connections across the injury site can now be used to elucidate the local circuits involved in this behaviour. The growth of axons across the injury in P7 lesioned opossums compared to lack of such growth in P28 lesioned opossums provides a system in which cellular-molecular differences between these two ages, differences that may account for the predominantly supraspinally mediated recovery that occurs in opossums lesioned at P7, may be studied.

## Supporting Information

Figure S1 **Representative gait traces from control, P7-injured and P28-injured opossums when tested as adults.** Opossums walked on a treadmill at 6 metres/min. Foot placements and lift-offs for each limb were manually plotted after viewing footage frame-by-frame. Green bar represents stance phase. Red line connects limb placements in the order in which they were placed. Each box represents a single frame (0.03 s) ***A:*** Control opossum; ***B:*** P7-injured opossum; ***C:*** P28-injured opossum.
(TIFF)

Video S1 **Treadmill footage sequence: control, P7-injured and P28-injured opossums tested as adult animals.** Video footage of opossums walking on a treadmill running at 6 metres/minute. Description of gait analysis can be found in Fig. S1.
(MP4)

Video S2 **Swimming footage sequence: control, P7-injured and P28-injured opossums tested as adults.** Video footage of opossums performing the swimming test and climbing onto the exit platform. Opossums were assessed on their ability to use their hindlimbs when swimming. Note the use of hindlimbs by P28-injured opossums once they touch the exit platform.
(MP4)

## References

[1] Saunders NR, Adam E, Reader M, Mollgard K (1989). Monodelphis domestica (grey short-tailed opossum): an accessible model for studies of early neocortical development.. Anat Embryol (Berl).

[2] Saunders NR, Balkwill P, Knott G, Habgood MD, Mollgard K (1992). Growth of axons through a lesion in the intact CNS of fetal rat maintained in long-term culture.. Proc Biol Sci.

[3] Treherne JM, Woodward SK, Varga ZM, Ritchie JM, Nicholls JG (1992). Restoration of conduction and growth of axons through injured spinal cord of neonatal opossum in culture.. Proc Natl Acad Sci U S A.

[4] Woodward SK, Treherne JM, Knott GW, Fernandez J, Varga ZM (1993). Development of connections by axons growing through injured spinal cord of neonatal opossum in culture.. J Exp Biol.

[5] Nicholls JG, Vischer H, Varga Z, Erulkar S, Saunders NR (1994). Repair of connections in injured neonatal and embryonic spinal cord in vitro.. Prog Brain Res.

[6] Saunders NR, Deal A, Knott GW, Varga ZM, Nicholls JG (1995). Repair and recovery following spinal cord injury in a neonatal marsupial (Monodelphis domestica).. Clin Exp Pharmacol Physiol.

[7] Saunders NR, Kitchener P, Knott GW, Nicholls JG, Potter A (1998). Development of walking, swimming and neuronal connections after complete spinal cord transection in the neonatal opossum, Monodelphis domestica.. J Neurosci.

[8] Wang XM, Basso DM, Terman JR, Bresnahan JC, Martin GF (1998). Adult opossums (Didelphis virginiana) demonstrate near normal locomotion after spinal cord transection as neonates.. Exp Neurol.

[9] Wang XM, Terman JR, Martin GF (1998). Regeneration of supraspinal axons after transection of the thoracic spinal cord in the developing opossum, Didelphis virginiana.. J Comp Neurol.

[10] Wang XM, Terman JR, Martin GF (1996). Evidence for growth of supraspinal axons through the lesion after transection of the thoracic spinal cord in the developing opossum Didelphis virginiana.. J Comp Neurol.

[11] Fry EJ, Stolp HB, Lane MA, Dziegielewska KM, Saunders NR (2003). Regeneration of supraspinal axons after complete transection of the thoracic spinal cord in neonatal opossums (Monodelphis domestica).. J Comp Neurol.

[12] Basso DM, Beattie MS, Bresnahan JC (1995). A sensitive and reliable locomotor rating scale for open field testing in rats.. J Neurotrauma.

[13] Robinson GA, Goldberger ME (1986). The development and recovery of motor function in spinal cats. I. The infant lesion effect.. Exp Brain Res.

[14] Howland DR, Bregman BS, Tessler A, Goldberger ME (1995). Development of locomotor behavior in the spinal kitten.. Exp Neurol.

[15] Stelzner DJ, Ershler WB, Weber ED (1975). Effects of spinal transection in neonatal and weanling rats: survival of function.. Exp Neurol.

[16] Weber ED, Stelzner DJ (1977). Behavioral effects of spinal cord transection in the developing rat.. Brain Res.

[17] Stelzner DJ, Weber ED, Prendergast J (1979). A comparison of the effect of mid-thoracic spinal hemisection in the neonatal or weanling rat on the distribution and density of dorsal root axons in the lumbosacral spinal cord of the adult.. Brain Res.

[18] Lane MA, Truettner JS, Brunschwig JP, Gomez A, Bunge MB (2007). Age-related differences in the local cellular and molecular responses to injury in developing spinal cord of the opossum, Monodelphis domestica.. Eur J Neurosci.

[19] Migliavacca A (1930). La régénération du système nerveax central avant et après la naissance.. Archives Italiennes de Biologie.

[20] Fadem BH, Trupin GL, Maliniak E, VandeBerg JL, Hayssen V (1982). Care and breeding of the gray, short-tailed opossum (Monodelphis domestica).. Lab Anim Sci.

[21] Kraus DB, Fadem BH (1987). Reproduction, development and physiology of the gray short-tailed opossum (Monodelphis domestica).. Lab Anim Sci.

[22] Vandeberg JL, Poole T, English P (1999). The laboratory opossum.. UFAW Handbook on the Management of Laboratory Animals. 7 ed.

[23] Basso DM, Beattie MS, Bresnahan JC (1996). Graded histological and locomotor outcomes after spinal cord contusion using the NYU weight-drop device versus transection.. Exp Neurol.

[24] Cheng H, Almstrom S, Gimenez-Llort L, Chang R, Ove Ogren S (1997). Gait analysis of adult paraplegic rats after spinal cord repair.. Exp Neurol.

[25] Deumens R, Jaken RJ, Marcus MA, Joosten EA (2007). The CatWalk gait analysis in assessment of both dynamic and static gait changes after adult rat sciatic nerve resection.. J Neurosci Methods.

[26] Koopmans GC, Deumens R, Honig WM, Hamers FP, Steinbusch HW (2005). The assessment of locomotor function in spinal cord injured rats: the importance of objective analysis of coordination.. J Neurotrauma.

[27] Oswald-Cruz E, Rocha-Miranda C (1968). The brain of the opossum *(Didelphis marsupialis)*: a cytoarchitectonic atlas in stereotaxic coordinates.

[28] Qin YQ, Wang XM, Martin GF (1993). The early development of major projections from caudal levels of the spinal cord to the brainstem and cerebellum in the gray short-tailed Brazilian opossum, Monodelphis domestica.. Brain Res Dev Brain Res.

[29] Lane MA (2004). The response of the developing nervous system to injury.

[30] Stolp HB, Ek CJ, Johansson PA, Dziegielewska KM, Bethge N (2009). Factors involved in inflammation-induced developmental white matter damage.. Neurosci Lett.

[31] Kunkel-Bagden E, Dai HN, Bregman BS (1993). Methods to assess the development and recovery of locomotor function after spinal cord injury in rats.. Exp Neurol.

[32] Courtine G, Song B, Roy RR, Zhong H, Herrmann JE (2008). Recovery of supraspinal control of stepping via indirect propriospinal relay connections after spinal cord injury.. Nat Med.

