# Supplementary figures and images for: Spontaneous Development of Full Weight-Supported Stepping after Complete Spinal Cord Transection in the Neonatal Opossum, Monodelphis domestica
Source: PLoS One. 2011 Nov 2;6(11):e26826. doi: 10.1371/journal.pone.0026826 (PMC3206848; doi:10.1371/journal.pone.0026826)

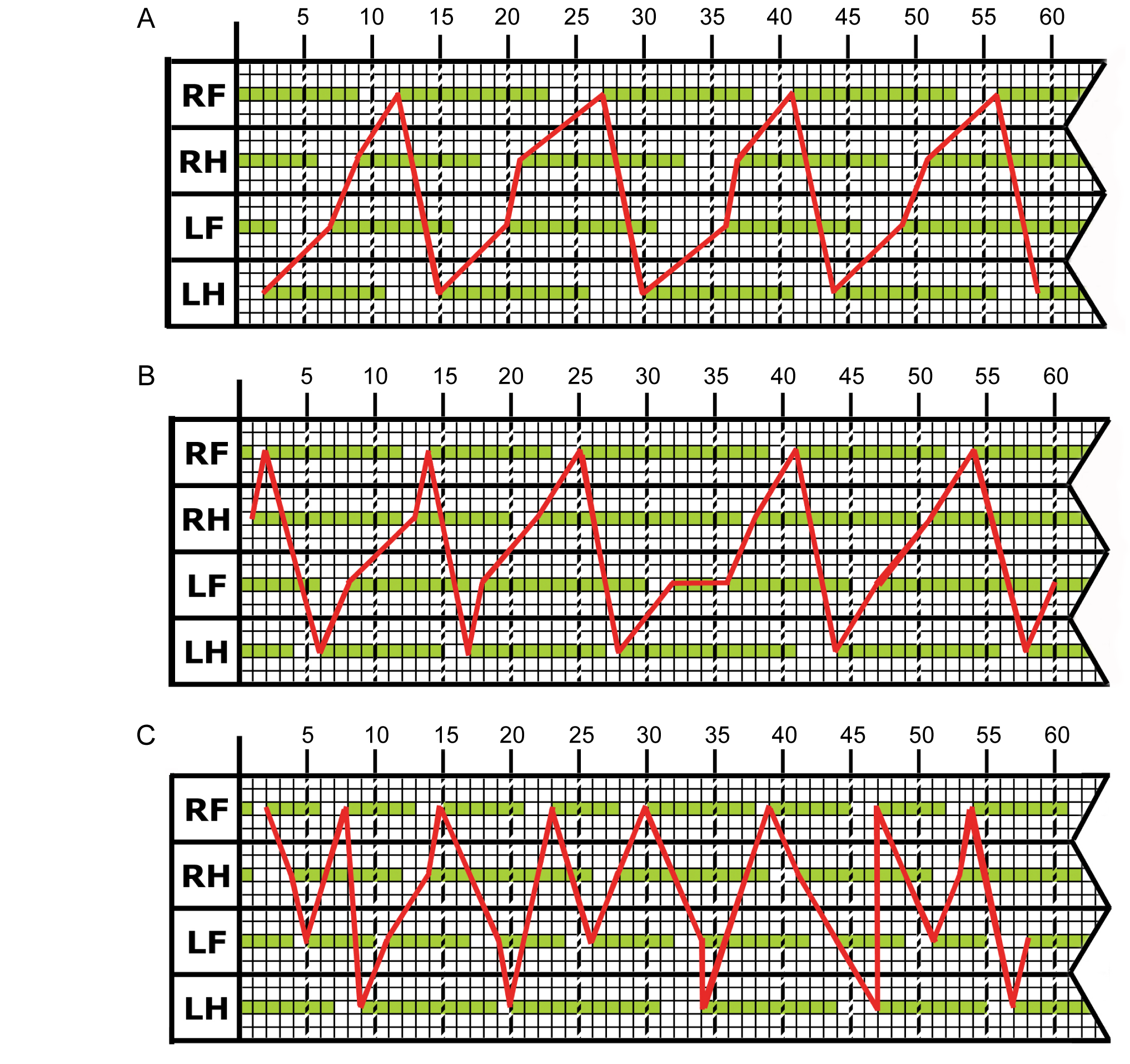

Supplement: Figure S1 — Representative gait traces from control, P7-injured and P28-injured opossums when tested as adults. Opossums walked on a treadmill at 6 metres/min. Foot placements and lift-offs for each limb were manually plotted after viewing footage frame-by-frame. Green bar represents stance phase. Red line connects limb placements in the order in which they were placed. Each box represents a single frame (0.03 s) A: Control opossum; B: P7-injured opossum; C: P28-injured opossum. (TIFF) [file pone.0026826.s001.tif]
